# Implementation of Guidelines on Family Involvement for Persons with Psychotic Disorders (IFIP): A Cluster Randomised Controlled Trial

**DOI:** 10.1007/s10488-023-01255-0

**Published:** 2023-02-16

**Authors:** Lars Hestmark, Maria Romøren, Kristin Sverdvik Heiervang, Kristiane Myckland Hansson, Torleif Ruud, Jūratė Šaltytė Benth, Irene Norheim, Bente Weimand, Reidar Pedersen

**Affiliations:** 1 Centre for Medical Ethics, University of Oslo, Postbox 1130, Blindern, 0318 Oslo, Norway; 2 Division of Mental Health Services, Akershus University Hospital, Lørenskog, Norway; 3 Center for Mental Health and Substance Abuse, Faculty of Health and Social Sciences, University of South-Eastern Norway, Drammen, Norway; 4 Institute of Clinical Medicine, University of Oslo, Oslo, Norway; 5 Institute of Clinical Medicine, Campus Ahus, University of Oslo , Oslo, Norway; 6 Health Services Research Unit, Akershus University Hospital, Lørenskog, Norway; 7 Division of Mental Health and Addiction, Vestre Viken Hospital Trust, Lier, Norway; 8 Faculty of Health Sciences, OsloMet Oslo Metropolitan University, Oslo, Norway

**Keywords:** Psychotic disorders, Family involvement, Family psychoeducation, Fidelity scale, Guideline implementation, Mental health services

## Abstract

Family involvement is part of the evidence-based treatment for persons with psychotic disorders, yet is under-implemented despite guideline recommendations. This study assessed whether an implementation support programme increased the adherence to guidelines on family involvement, compared to guideline/manual only. In a cluster randomised design, community mental health centre units in South-East Norway went through stratified allocation to the experimental (n = 7) or control (n = 7) arm. Experimental clusters received an implementation support programme including clinical training and supervision, appointing a family coordinator and an implementation team, a toolkit, and fidelity measurements at baseline, 12, 18, and 24 months with on-site feedback and supervision. Control clusters received no such support and had fidelity measurements at baseline and 24 months without feedback. During fidelity measurements, adherence to the guidelines was measured with the basic family involvement and support scale, the general organizational index, and the family psychoeducation fidelity scale, the latter being the primary outcome. The scales consist of 12–14 items rated from 1 to 5. Data was analysed with an independent samples t-test, linear mixed models, and a tobit regression model. At 24 months, the mean scores were 4.00 or higher on all scales in the experimental arm, and the increase in adherence to the guidelines was significantly greater than in the control arm with p-values < 0.001. Large-scale implementation of guidelines on family involvement for persons with psychotic disorders in community mental health centres may be accomplished, with substantial implementation support.

**Trial Registration**: ClinicalTrials.gov Identifier NCT03869177. Registered 11.03.19.

**Supplementary Information:**

The online version contains supplementary material available at 10.1007/s10488-023-01255-0.

## Introduction

Family involvement is a key element of the evidence-based treatment for persons with psychotic disorders (F20–29 in ICD-10). Its fundamental role is supported by well-documented beneficial effects for patients and relatives (Bighelli et al., 2021; Bird et al., 2010; Claxton et al., 2017; Hasan & Jaber, 2019; Lobban et al., 2013; Ma et al., 2018; Pharoah et al., 2010; Pilling et al., 2002; Pitschel-Walz et al., 2001; Rodolico et al., 2022; Sin et al., 2017; Yesufu-Udechuku et al., 2015), but also rests on firm moral and legal foundations.

Family psychoeducation (FPE) is a structured family intervention that includes separate alliance sessions with patient and relative(s) followed by joint psychoeducative sessions, communication skills exercises, and problem solving sessions (Lucksted et al., 2012). Based on a synthesis of the scientific literature, clinical practice guidelines worldwide recommend such family interventions as a first-line treatment during all stages of psychotic disorders (Dixon et al., 2010; Galletly et al., 2016; Gühne et al., 2015; Kuipers et al., 2014). Even so, the implementation of family interventions in mental health services appears generally poor and unsystematic, with few patients and relatives receiving such interventions (Bucci et al., 2016; Hestmark et al., 2021; Rummel-Kluge et al., 2006). Studies also indicate that even the most basic forms of family involvement, cooperation, and support are offered irregularly (Hestmark et al., 2021; Vermeulen et al., 2015; Weimand et al., 2011). This highlights the need for implementation research with a focus on both basic and advanced levels of family involvement.

Family interventions are not the only evidence-based practices (EBPs) that suffer from underuse in mental health care (Torrey et al., 2001). General barriers that hinder the adoption of EBPs or clinical practice guidelines in the health services include a lack of leadership commitment and prioritisation, conflicting professional views, lack of resources, structure, training, and supervision (Bucci et al., 2016). Yet, the implementation of family involvement practices in mental health care faces additional and particular obstacles of a clinical, ethical, cultural, and historical nature. Examples include biomedical paradigms where family involvement is not considered treatment, historical paradigms where relatives are considered a significant cause of the illness, and ethical dilemmas concerning patient autonomy and the duty of confidentiality (Eassom et al., 2014; Landeweer et al., 2017; Szmukler & Bloch, 1997). Thus, a systematic effort to implement family involvement in mental health services should include strategies to address both general and particular barriers.

Implementation strategies frequently used in mental health services research include training and supervision, toolkits and educational material, local or regional support teams, and some form of quality or fidelity monitoring (Menear & Briand, 2014). Fidelity is a central implementation outcome, assessing whether the intervention was delivered and implemented as prescribed (Proctor et al., 2011). The rationale is that the implementation of core elements of EBPs, previously tested through rigorous research designs, will generate similar outcomes. Fidelity measurements may also enable researchers to distinguish between failure of the intervention and failure of implementation (Bond & Drake, 2020). Previous fidelity-based studies on the implementation of FPE have been either experimental non-randomised trials (Kealey et al., 2015; McHugo et al., 2007), or unable to demonstrate significant increases in fidelity (Ruud et al., 2021).

In 2017, the Norwegian Directorate of Health issued national recommendations on family involvement and support in the health- and care services, based on legal regulations, research evidence, ethical considerations, and discussions between key stakeholders and experts (Norwegian Directorate of Health, 2017). These general recommendations supplement the clinical practice guidelines that concern family interventions specifically in the treatment of psychotic disorders (Norwegian Directorate of Health, 2013). We refer to the general and specific guidelines collectively as ‘the national guidelines’. The results from a systematic baseline survey, of family involvement practices in participating clinical sites, suggest that the level of implementation of these guidelines in Norwegian community mental health centres (CMHCs) was generally low (Hestmark et al., 2021).

The purpose of the ‘Implementation of Family Involvement for persons with Psychotic disorders’ (IFIP) trial was to implement selected recommendations from the national guidelines in Norwegian CMHCs (Hestmark et al., 2020). With a comprehensive Implementation Support Programme (ISP), the project sought to implement a combination of basic and advanced levels of family involvement, using both general and specific implementation strategies to address barriers on multiple levels. The aim of this article is to answer the following research question: Did the IFIP ISP lead to an increased adherence to the national guidelines, compared to guideline/manual only?

## Methods

This article conforms to the ‘Consolidated Standards of Reporting Trials (CONSORT) statement 2010: extension to cluster randomised trials’ (Campbell et al., 2012) (Supplementary file 1).

### Trial Design, Sample Size, and Participating Clinical Sites

The IFIP trial employed a cluster randomised controlled design. A cluster was defined as one or more CMHC outpatient units that had the main responsibility for long-term treatment of patients with psychotic disorders in a discrete catchment area. There were no further eligibility criteria for clusters. The design was appropriate to analyse differences in implementation outcomes between experimental and control conditions, but also critical to avoid contamination in the sub study on patients’ and relatives’ outcomes (Hestmark et al., 2020).

Adherence to the national guidelines was assessed through fidelity measurements. The unit of analysis was the cluster, and fidelity outcomes pertain to the cluster level. When calculating the sample size, we assumed a mean difference in fidelity scores of 1.82 with a standard deviation of 0.80, after 18 months of implementation support. These numbers were based on the results from two previous implementation studies using the family psychoeducation fidelity assessment (FPE) scale (Kealey et al., 2015; McHugo et al., 2007), which therefore must be regarded as the primary outcome, although the remaining scales are of equal importance. For a two-sided Independent samples t-test, with 5% significance level and 80% power, we estimated that four clusters in each arm were required to show that implementation support leads to a significant increase in adherence. Since the IFIP trial also assessed outcomes for patients and relatives, it required seven clusters in each arm to secure adequate power, taking the number of potential participants and the cluster effect into account (Hestmark et al., 2020).

All the 16 CMHCs in five counties of the South-Eastern Norway Regional Health Authority were invited to participate in the trial, and 15 clinical sites from 12 CMHCs in 6 health trusts agreed to participate during summer/fall 2018. These 12 CMHCs together serve approximately 25% of the Norwegian population. Among the remaining CMHCs, the principal reason given for non-participation was a lack of capacity to take part in a research project. The participating clinical sites included various adult service types, such as assertive outreach teams, early intervention units, dual diagnosis teams, as well as mixed or specialised outpatient clinics. Their clients were 18 years or above, and included both patients with recently diagnosed and chronic psychotic disorders. A detailed account of the participating clinical sites and their baseline fidelity scores has been published (Hestmark et al., 2021). Each site corresponds to one cluster, except for two collaborating sites that were merged to get an even number of clusters for randomisation. There was no drop-out of clusters during the trial, neither from the intervention in the experimental arm, nor from analysis in either arm.

### Randomisation

**Fig. 1:**
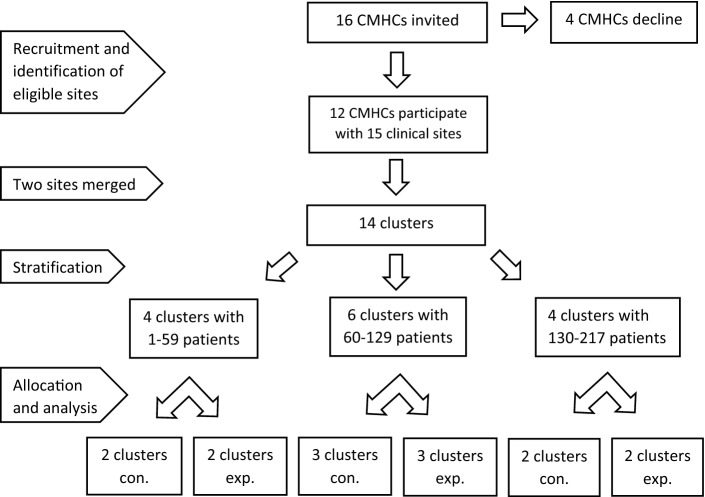
Flow diagram of the recruitment, stratification, allocation, and analysis of clusters in the IFIP trial. *CMHC* Community mental health centre, *Con* control, *Exp* experimental

Figure 1 illustrates the flow of clusters through recruitment, allocation, and analysis. The project group generated a sequence by ranking the clusters according to their number of patients with psychotic disorders. The clusters were then stratified into three even-numbered blocks, and within each block, they were randomised to the experimental or control arm with an allocation ratio of 1:1. An independent and blinded statistician performed the allocation by drawing 14 numbers with the Microsoft Excel RAND function.

### Intervention

The project group developed the IFIP intervention to operationalise the national guidelines. An elaborate description of the intervention and its development can be found in the study protocol (Hestmark et al., 2020). A qualitative exploration of the implementation process, in terms of barriers and facilitators, has also been published (Hansson et al., 2022).

**Fig. 2:**
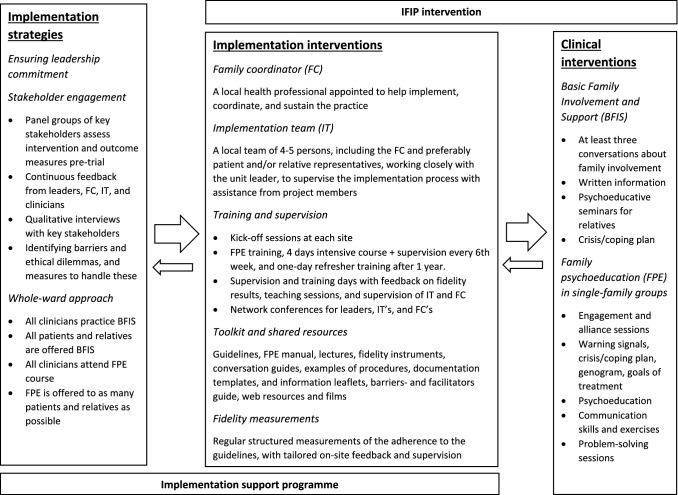
The IFIP intervention and Implementation support programme (ISP)

Figure 2 displays the implementation strategies, implementation interventions, and clinical interventions of the IFIP trial, and how these were connected through continuous feedback loops. It also illustrates how ‘The IFIP intervention’ refers to both the implementation- and clinical interventions of the trial, whereas ‘The implementation support programme’ (ISP) refers to all the strategies and activities intended to support the implementation of the clinical interventions. The experimental clusters received the ISP for 18 months, whereas the control clusters did not receive such support during this period.

The ISP was based on the seminal work of the National Evidence-Based Practices (NEBP) project (Bond et al., 2009a; McHugo et al., 2007), and on a recent Norwegian RCT (Ruud et al., 2021). We adopted elements such as the constitution of a local implementation team, regular fidelity measurements with tailored feedback and on-site supervision, kick-off sessions, training and supervision in FPE, a toolkit, a local programme coordinator (family coordinator), interviews with leaders and practitioners, mapping of barriers and facilitators, and a particular emphasis on leadership commitment. Each clinical site had a regular contact person from the research team, developing a continuous working relationship with the local leader(s), implementation team, and family coordinator. The researchers who measured fidelity also conducted the supervision of the local implementation teams, using the recent fidelity results to identify areas for improvement and make detailed 6-month plans for implementation activities, as part of on-site ‘Training and supervision days’. The latter also included plenary sessions with all the clinicians at the unit, with feedback on fidelity results, presentation and discussion of goals set by the implementation team, training in how to handle the duty of confidentiality during family involvement, and presentations of relevant tools. Training and supervision in FPE was provided by The Early Intervention in Psychosis Advisory Unit for South East Norway. The role of the family coordinator was comprehensive and intended as a permanent part of the organisation to promote sustainability of the new practice.

In addition, the IFIP trial employed several distinct implementation strategies: Stakeholder engagement inspired by a responsive evaluation approach (Abma, 2006), a whole-ward approach (Sævareid et al., 2019), and the combination of FPE and Basic Family Involvement and Support (BFIS) (Hestmark et al., 2020, 2021). Throughout the trial, we interviewed key stakeholders and received feedback from the participating units, as part of a responsive process evaluation, to adjust the implementation strategy and effort. This interactive approach was further employed to investigate key barriers and ethical dilemmas, and to identify possible solutions and facilitators for implementation (Hansson et al., 2022).

The whole-ward approach was intended to alter the culture and clinical modus operandi of entire health care units (Sævareid et al., 2019). Since awareness, attitudes, and clinical skills varied considerably when it came to family involvement, we recommended that all clinicians should receive FPE training to gain a shared understanding and appreciation of its benefits (Mottaghipour et al., 2006). A second feature of this approach was the recommendation that all clinical personnel should acquire BFIS skills, and provide such services to all patients with psychotic disorders and their relatives. The diffusion of awareness, competence, and skills was also intended as a sustainability measure, to render the new practice less vulnerable to staff turnover. By promoting BFIS, we sought to increase the frequency of contact between relatives and health personnel, potentially leading to increased levels of FPE as well.

There were no specific qualifications required for being appointed as a family coordinator or implementation team member, or for delivering BFIS and FPE, other than the training and supervision offered as part of the trial.

### Instruments

We employed three fidelity scales to assess the adherence to the national guidelines. The scales consist of 12–14 items rated from 1 to 5, where 1 equals no implementation and 5 equals full implementation.

To measure basic family involvement and support (BFIS), the project group developed a new 14-item fidelity scale with two subscales. One subscale (BFIS-S) examines structure, content, and implementation, while the other (BFIS-P) measures ‘penetration rate’. The latter term means the percentage of eligible patients and/or relatives that receive a particular intervention. A description of the development process, content, and psychometric properties of the BFIS scale has been published (Hestmark et al., 2021). Due to limited time for piloting, some items were removed or changed after the baseline data were collected, resulting in minor adjustments of the baseline scores.

The 14-item family psychoeducation fidelity assessment (FPE) scale rates the practice and content of FPE, whereas the 12-item general organizational index (GOI) scale measures the individualisation, quality improvement, program philosophy, and penetration rate of FPE. Previous studies report acceptable psychometric properties for both scales (Bond et al., 2009b; Heiervang et al., 2020; Joa et al., 2020; Kealey et al., 2015). An average score of 4 or above on either scale denotes adequate implementation, while scores below 4 indicate moderate to low implementation. Sites that did not offer FPE were scored 1 on all items on both scales. Item 7: ‘prodromal signs’ in the FPE scale was omitted, since the participating sites rarely treated patients with prodromal or ultra-high risk states.

### Data Collection

The timeline in Supplementary file 2 shows the intervals between fidelity measurements in the experimental arm. The official start of the implementation period was 6 months after the baseline fidelity measurements, with the first follow-up measurements 6 months later and then every 6th month throughout the trial. Fidelity assessments in the control arm were only performed at baseline and 24 months. When measuring fidelity at baseline and 12 months, the assessors visited the clinical sites. However, because of the coronavirus pandemic, we had to employ a digital video conference platform for some of the measurements at 18 months, and all of the measurements at 24 months.

At each site, two researchers measured fidelity by conducting structured interviews with leaders, clinicians, and resource persons, and by examining written material such as procedures and information leaflets. They performed 2–5 separate interviews of 1–1.5 h length. Usually the head of department was interviewed individually, whereas those in other participant categories were interviewed in groups of 2–6 persons. Verbal informed consent was obtained from all participants prior to the interviews. The two fidelity assessors first scored all items independently and then resolved any discrepancies to reach a consensus score for each item. Where clusters consisted of subunits with differing clinical approaches and patient populations, their average scores were recorded. The two experimental sites that were merged to a single cluster were scored separately throughout the trial, and their average scores were calculated at each time point as the cluster scores. We solely assessed the sites’ practice towards patients with psychotic disorders and their relatives. At each time point, we also recorded the percentage of patients with psychotic disorders that had received or were receiving FPE, based on administrative data. When calculating these percentages, the denominator only included patients currently receiving treatment at the clinical unit.

The assessors, and the pairing of them, varied across both sites and time points. None of the five researchers who assessed fidelity throughout the trial were employees of the clinical sites in the study. At each time point, the fidelity assessors prepared a detailed report for the respective site to complement the scores. Scores and reports were made available to the sites in the experimental arm, but not to the sites in the control arm, to reduce the influence of fidelity assessments on their practice during the implementation period. Due to obvious changes in the practice and organisation of experimental sites, and the fact that researchers provided implementation support, it was impossible to blind the assessments.

### Data Analyses

To assess interrater reliability (IRR), we calculated the intra-class correlation coefficient (ICC) for each scale’s total mean fidelity, using a one-way random effects analysis of variance model for agreement between two assessors.

In accordance with the premises of the sample size calculation, difference between experimental and control arms in change on the FPE scale (primary outcome) from baseline to 24 months, was assessed by an Independent samples t-test. The results were presented as mean difference with corresponding 95% confidence interval (CI), p-value and effect size (Cohen’s d) with 95% CI.

Differences between the experimental and control arms in change on the FPE scale, the GOI scale, the BFIS scale, and its subscales BFIS-S and BFIS-P were assessed by linear mixed models (LMMs) with random intercepts for clusters. Random effects for Health trust were also considered, but skipped, as the model fit was not improved according to Bayes Information Criterion. To account for potentially non-linear trend through four time points in the experimental arm and model linear trend in the control arm with measurements at two time points only, we estimated the following model with respect to fixed effects:

$${\text{y}} = \beta_{0} + \beta_{{1}} ^{*}{\text{Group}} + \beta_{{2}} ^{*}{\text{t}}_{{{12}}} ^{*}{\text{Group}} + \beta_{{3}} ^{*}{\text{t}}_{{{18}}} ^{*}{\text{Group}} + \beta_{{2}} ^{*}{\text{t}}_{{{24}}} + \beta_{{5}} ^{*}{\text{t}}_{{{24}}} ^{*}{\text{Group}},$$

where t_12_, t_18_ and t_24_ are dummies for time, Group is dummy for group (0 for control and 1 for experimental group), and t_12_*Group, t_18_*Group and t_24_*Group are interactions between time dummies and group dummy. Differences in change in the percentage of patients receiving FPE were analysed with a tobit regression model for longitudinal data with the same fixed and random effects as above. A priori planned adjustment for the stratification variable was explored.

Post hoc analyses, not planned a priori, were performed to assess within-group changes as well as between-group differences and between-group differences in changes. The results were presented as observed means and standard deviations (SDs) and mean changes and differences with corresponding 95% CIs and p-values as well as effect sizes (Cohen’s d) with 95% CIs estimated from LMM or tobit model. The results with p-values below 0.05 were considered statistically significant. No adjustment for multiple testing was performed, as the post hoc analyses were of exploratory nature. Standard residual diagnostic was performed. Data analyses were performed using IBM SPSS statistics version 28 and STATA version 17.

## Results

Concerning IRR, we calculated an ICC of 0.99 for mean total fidelity of the BFIS scale, based on all 46 fidelity measurements. With regard to the FPE scale, we estimated an ICC of 0.99 for mean total fidelity, and the ICC of the GOI scale was 0.99. When calculating ICC for the GOI and FPE scales, we only included the 34 fidelity measurements where the unit in question offered FPE.

Mean difference between the study arms in change on the FPE scale from baseline to 24 months was 2.69 with 95% CI (0.67; 4.71), p = 0.013, and effect size 1.55 (0.32; 2.75).

The results of the linear mixed models and the tobit regression model are reported in Table 1. It shows that the increase in fidelity scores on all scales and BFIS subscales from baseline to 24 months was significantly larger for experimental clusters than control clusters with p-values < 0.001. The difference in change in the percentage of patients receiving FPE was also significant with p = 0.01. Adjustment for the stratification variable did not affect the results (Supplementary file 3).

**Table 1:** Results of linear mixed models and tobit regression model for difference in change between the experimental and control arm

	BFIS mean^b^		BFIS-S mean^b^		BFIS-P mean^b^		GOI mean^b^		FPE scale mean^b^		FPE % mean^c^	
	RC (SE)	p-value	RC (SE)	p-value	RC (SE)	p-value	RC (SE)	p-value	RC (SE)	p-value	RC (SE)	p-value
Intercept	2.25 (0.16)	< 0.001	1.66 (0.16)	< 0.001	2.59 (0.17)	< 0.001	1.77 (0.21)	< 0.001	2.87 (0.47)	< 0.001	1.19 (3.88)	0.760
Group^a^	0.22 (0.22)	0.320	0.09 (0.23)	0.705	2.29 (0.25)	0.232	0.04 (0.30)	0.889	− 0.14 (0.67)	0.837	2.92 (5.41)	0.589
T12 x Group	1.01 (0.14)	** < 0.001**	1.71 (0.14)	** < 0.001**	0.61 (0.16)	** < 0.001**	2.12 (0.24)	** < 0.001**	1.18 (0.51)	**0.020**	10.61 (2.67)	** < 0.001**
T18 x Group	1.30 (0.14)	** < 0.001**	1.93 (0.14)	** < 0.001**	0.95 (0.16)	** < 0.001**	2.22 (0.24)	** < 0.001**	1.71 (0.51)	**0.001**	10.03 (2.67)	** < 0.001**
T24	0.11 (0.14)	0.407	− 0.03 (0.14)	0.836	0.19 (0.16)	0.245	− 0.37 (0.24)	0.125	− 0.94 (0.51)	0.062	− 1.27 (2.83)	0.653
T24 x Group	1.41 (0.19)	** < 0.001**	2.29 (0.19)	** < 0.001**	0.93 (0.23)	** < 0.001**	2.56 (0.34)	** < 0.001**	2.69 (0.72)	** < 0.001**	10.00 (3.88)	**0.010**

Statistically significant *p*-values are given in bold

^a^Control arm—reference

^b^Linear mixed model

^c^Tobit regression model for longitudinal outcome

**Fig. 3:**
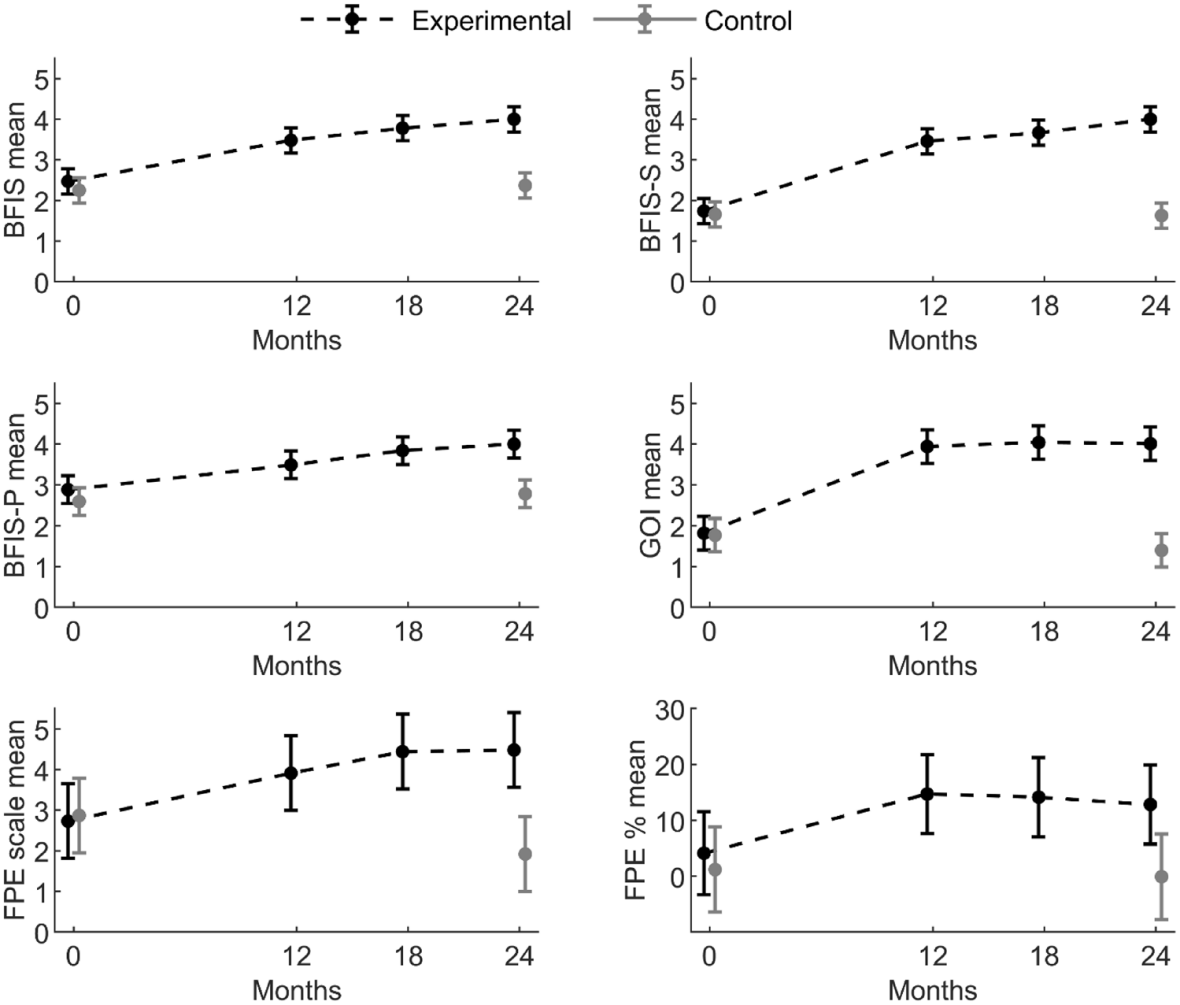
Mean fidelity scores with 95% CIs in experimental and control clusters from baseline to 24 months. Results of linear mixed models and tobit regression model

The descriptive statistics reported in Table 2 show that the mean scores among experimental clusters at 24 months were ≥ 4.00 on all scales, whereas the corresponding mean scores in the control arm were < 3.00. Estimated mean fidelity scores at each time point, with 95% CIs, are depicted for both arms in Fig. 3.

**Table 2:** Descriptive statistics for outcome variables and results of post hoc analysis from linear mixed models and tobit regression model for between-arm differences

Time point	Experimental arm Mean (SD)^1^	Control arm Mean (SD)^1^	Experimental vs. control arm Mean difference (95% CI)^2^
BFIS mean			
0	2.47 (0.63)	2.25 (0.16)	0.22 (− 0.21; 0.65)
12	3.48 (0.56)		
18	3.78 (0.51)		
24	4.00 (0.37)	2.37 (0.41)	1.63 (1.20; 2.06)
BFIS-S mean			
0	1.74 (0.60)	1.66 (0.19)	0.09 (− 0.36; 0.53)
12	3.46 (0.59)		
18	3.67 (0.54)		
24	4.00 (0.37)	1.63 (0.47)	2.37 (1.93; 2.82)
BFIS-P mean			
0	2.88 (0.70)	2.59 (0.24)	0.29 (− 0.19; 0.77)
12	3.49 (0.61)		
18	3.84 (0.55)		
24	4.00 (0.43)	2.78 (0.41)	1.22 (0.74; 1.80)
GOI mean			
0	1.82 (0.91)	1.77 (0.76)	0.04 (− 0.54; 0.62)
12	3.94 (0.27)		
18	4.04 (0.23)		
24	4.01 (0.22)	1.40 (0.69)	2.60 (2.02; 3.19)
FPE scale mean			
0	2.73 (1.77)	2.87 (1.76)	− 0.14 (− 1.44; 1.17)
12	3.91 (1.21)		
18	4.44 (0.21)		
24	4.48 (0.22)	1.92 (1.58)	2.55 (1.25; 3.86)
FPE % mean			
0	6.76 (6.88)	4.09 (4.62)	2.92 (− 7.68; 13.52)
12	14.71 (14.10)		
18	14.14 (9.43)		
24	12.84 (11.92)	2.99 (4.62)	12.93 (2.52; 23.34)

^1^Observed mean and standard deviation (SD)

^2^Mean difference and 95% confidence interval (CI) estimated from linear mixed model or tobit regression for longitudinal data (for FPE % mean)

Table 3 displays the post hoc analyses of mean fidelity changes within arms and the mean differences in change between arms for each time interval. The changes in fidelity between baseline and 24 months in the control arm were not significant on any scale. In the experimental arm, the changes between baseline and 12, 18, and 24 months were significant on all scales and subscales. The differences in fidelity changes between experimental and control arms between baseline and 24 months were all significant, and the corresponding effect sizes were substantial.

**Table 3:** Mean changes and between-group differences in changes with 95% CIs. Results of post hoc analysis from linear mixed models and tobit regression model

Interval	Experimental arm		Control arm		Experimental vs. control arm		
	Mean change (95% CI)	p-value	Mean change (95% CI)	p-value	Mean change (95% CI)	p-value	Cohen’s d (95% CI)
BFIS mean							
0–12 0–18 0–24 12–18 12–24 18–24	1.01 (0.74; 1.28) 1.30 (1.03; 1.57) 1.52 (1.25; 1.79) 0.29 (0.02; 0.56) 0.52 (0.25; 0.79) 0.22 (− 0.05; 0.49)	< 0.001 < 0.001 < 0.001 0.033 < 0.001 0.106	0.11 (− 0.16; 0.38)	0.407	1.41 (1.03; 1.79)	< 0.001	3.41 (1.69; 5.13)
BFIS-S mean							
0–12 0–18 0–24 12–18 12–24 18–24	1.71 (1.44; 1.98) 1.93 (1.66; 2.20) 2.26 (1.99; 2.53) 0.21 (− 0.06; 0.48) 0.54 (0.27; 0.81) 0.33 (0.06; 0.60)	< 0.001 < 0.001 < 0.001 0.120 < 0.001 0.017	− 0.03 (− 0.30; 0.24)	0.836	2.29 (1.90; 2.67)	< 0.001	5.40 (3.00; 7.80)
BFIS-P mean							
0–12 0–18 0–24 12–18 12–24 18–24	0.61 (0.29; 0.93) 0.95 (0.63; 1.27) 1.12 (0.80; 1.44) 0.34 (0.02; 0.66) 0.51 (0.19; 0.83) 0.17 (− 0.15; 0.49)	< 0.001 < 0.001 < 0.001 0.036 0.002 0.310	0.19 (− 0.13; 0.51)	0.245	0.93 (0.48; 1.38)	< 0.001	2.03 (0.70; 3.35)
GOI mean							
0–12 0–18 0–24 12–18 12–24 18–24	2.12 (1.65; 2.60) 2.22 (1.75; 2.69) 2.19 (1.72; 2.66) 0.10 (− 0.38; 0.57) 0.07 (− 0.41; 0.54) − 0.03 (− 0.50; 0.44)	< 0.001 < 0.001 < 0.001 0.687 0.781 0.901	− 0.37 (− 0.84; 0.10)	0.125	2.56 (1.89; 3.23)	< 0.001	4.60 (2.49; 6.72)
FPE scale mean							
0–12 0–18 0–24 12–18 12–24 18–24	1.18 (0.19; 2.17) 1.71 (0.72; 2.70) 1.75 (0.76; 2.74) 0.53 (− 0.46; 1.52) 0.57 (− 0.42; 1.56) 0.04 (− 0.95; 1.03)	0.020 0.001 0.001 0.293 0.260 0.939	− 0.94 (− 1.94; 0.05)	0.062	2.69 (1.29; 4.09)	< 0.001	2.16 (0.80; 3.52)
FPE % mean							
0–12 0–18 0–24 12–18 12–24 18–24	10.6 (5.4; 15.8) 10.0 (4.8; 15.3) 8.7 (3.5; 14.0) − 0.6 (− 5.3; 4.1) − 1.9 (− 6.6; 2.8) − 1.3 (− 6.0; 3.4)	< 0.001 < 0.001 0.001 0.812 0.436 0.588	− 1.3 (− 6.8; 4.3)	0.653	10.0 (2.4; 17.6)	0.010	1.00 (− 0.12; 2.12)

At baseline, 4 of 7 clusters in both arms offered FPE. However, at 24 months, all of the clusters in the experimental arm offered FPE, while only 2 clusters in the control arm did so. Table 2 displays how the mean percentage of patients with psychotic disorders, previously or currently receiving FPE, approximately doubled from 6.76 to 12.84% in the experimental arm, whereas it fell from 4.09 to 2.99% in the control arm. Post hoc analyses showed that the changes between baseline and 12, 18, and 24 months in the experimental arm were all significant, and the difference in change between arms from baseline to 24 months was also significant with p = 0.01.

## Discussion

The results show that the ISP had a significant and substantial effect on the adherence to the national guidelines in participating clusters, compared to manual/guideline only. At 24 months, the mean scores on all fidelity scales were four or higher in the experimental arm, suggesting adequate to excellent levels of implementation.

Structural elements of the BFIS scale such as implementation team, family coordinator, and procedures for family involvement were implemented during the first 6 months of the implementation period in the experimental arm, as demonstrated by the sharp rise in BFIS-S scores. By comparison, the BFIS-P scores increased progressively throughout the trial, probably reflecting that time is required for organisational and procedural changes to reach patients and relatives (Bond et al., 2009a, b; McHugo et al., 2007).

At 24 months, all experimental sites offered FPE with adequate fidelity (≥ 4) and a mean score of 4.48. The progressive nature of the FPE model probably explains the gradual increase in scores in the experimental arm. The success rate is good compared to previous studies, which report mean scores of 3.30–4.00 and 39–50% of sites reaching adequate fidelity after 18–24 months of implementation support (Bond et al., 2009a, b; Kealey et al., 2015; Ruud et al., 2021). However, these studies experienced high rates of discontinuation or unsuccessful implementation. Similarly to previous studies (Kealey et al., 2015; McHugo et al., 2007), the major increase in FPE fidelity happened in the first 12 months of the implementation period.

The GOI scale was used to investigate critical implementation factors beyond fidelity (Heiervang et al., 2020). A mean score across sites of 4.01, with 71% of sites reaching an adequate mean score (≥ 4) at 24 months constitute excellent results, compared to previous studies who report mean scores of 2.99–4.10 and 18–50% of sites reaching adequate levels after 12–24 months of implementation support for Illness Management and Recovery (Egeland et al., 2017; Heiervang et al., 2020; Salyers et al., 2009).

The substantial improvements in FPE fidelity and GOI scores in the experimental arm were not accompanied by large increases in the penetration rate of FPE. This might be related to capacity issues, the relatively short observation time, and the coronavirus pandemic (see below). By not including discharged patients who had received FPE, the numbers may also systematically underestimate the effort of the clinical sites. In contrast, the mean score on the BFIS-P subscale indicates that the penetration rate of basic family involvement practices rose to 60–80% across items in the experimental arm at 24 months. BFIS practices are less time-consuming than FPE and were usually implemented as standardised procedures towards all patients at the clinical sites, which may explain some of the difference in penetration rate. When calculating the penetration rates, we assumed that all patients with psychotic disorders were eligible for BFIS and FPE, which probably is an overestimation, particularly with regard to FPE (Haahr et al., 2021).

Similar to the implementation model of the NEBP project (Bond et al., 2009a, b), a central strategy was to use the fidelity scores actively to guide the implementation process in experimental sites, where the fidelity assessors supervised the local leader(s), implementation team, and family coordinator. Qualitative data indicate that this external support was a critical facilitator for implementation (Hansson et al., 2022).

The IFIP implementation strategy also differed from those of previous multi-centre fidelity-based studies on the implementation of FPE (Kealey et al., 2015; McHugo et al., 2007; Ruud et al., 2021). Implementing BFIS alongside FPE may have reinforced the adoption of both by the clinical sites. Introducing routines for early and systematic contact with relatives of all patients with psychotic disorders, by all clinicians, may have lowered the threshold for initiating advanced levels of family involvement, such as FPE (Hansson et al., 2022; Mottaghipour & Bickerton, 2005). By only targeting patients with psychotic disorders, and implementing single-family psychoeducation groups rather than multi-family groups or both, the project aimed to simplify the implementation- and recruitment processes for the sites.

The first coronavirus pandemic lockdown in Norway began approximately 2 months before the fidelity measurements at 18 months. The consequent lack of newly started FPE groups in the last 7–8 months of the implementation period contributed to the dip in FPE penetration rate seen at 18 and 24 months. Fidelity scores did not appear to be similarly affected, but it is difficult to ascertain whether the results could have been different. The lack of data points at 12 and 18 months in the control arm makes it harder to assess the influence of such external factors, but it is likely that the respective arms were affected to the same degree.

### Strengths and Limitations

As a pragmatic cluster randomised trial in a real-world setting, with clinical sites covering 25% of the Norwegian population, the findings may be considered robust and relevant to similar implementation efforts in the health services. To our knowledge, the IFIP trial is the first large-scale effort to implement basic family involvement practices in CMHCs, and the BFIS scale is the first instrument to assess such practices systematically.

In terms of external validity, our results describe practices towards a specific patient group and their relatives, in a particular clinical, geographical, and cultural context. Still, the generic character of many of the interventions and implementation strategies used suggests that these may be suitable in other clinical settings as well. By providing continuous feedback on the results (formative assessment), the external validity of the findings is limited to interventions that employ a similar implementation strategy (Lilford et al., 2009).

The study design could have been more suitable to evaluate the implementation strategy, if there was a ‘placebo’ implementation strategy in the control arm. However, this would have resulted in contamination of the sub study on patients’ and relatives’ outcomes. Since participants and researchers could not be blinded, there is a possibility that the sites’ allocation status influenced both the performance and evaluation of the respective arms. It was a deliberate and pragmatic decision to have the fidelity reviewers provide implementation support and supervision, because the insights gained through fidelity measurements enabled them to tailor the supervision to the respective unit’s needs. However, one could argue that they consequently assessed some of the results of their own effort, which introduced a risk of experimenter bias. This is most relevant when considering the results measured with the GOI scale and the BFIS subscale that examined structure, content, and implementation (BFIS-S). Both scales contain structural, procedural, and organisational elements, which level of implementation was influenced by the fidelity assessors through their supervision of the implementation teams. Yet, many of these elements are less susceptible to experimenter bias, because they are less open to interpretation. Examples include whether or not units had written information, procedures on family involvement, appointed a family coordinator, constituted an implementation team, or the percentage of clinical staff with FPE training. Since training and supervision in FPE was provided by an independent organisation, which had nothing to do with fidelity assessments, the results measured with the FPE scale (primary outcome) were not subject to a similar risk of experimenter bias.

By removing or altering a few elements of the BFIS scale after the baseline measurements, we potentially risked introducing bias and overestimating the intervention effect, if elements were removed that appeared hard to implement. However, the elements removed were covered by the other scales and the elements altered were generally made stricter and more specific. Fidelity raters did not observe FPE sessions, interview service users, or assess randomly selected patient records, all of which could have increased the validity of our findings. Concerning predictive validity, the present paper does not report on patients’ and relatives’ outcomes, but such data will be analysed and reported on later as part of the trial.

### Implications

The findings of the IFIP trial can and should be employed to scale up family involvement practices for persons with psychotic disorders in CMHCs. Research is needed on the sustainability of family involvement practices, on methods to scale up efficiently, on implementation for other patient groups with severe mental illness, and on implementation in other health- and care contexts, such as inpatient facilities and municipal health services.

## Conclusion

This study demonstrates that large-scale implementation of guidelines on family involvement for persons with psychotic disorders in CMHCs may be accomplished, with substantial implementation support combining general and specific implementation strategies.

## Supplementary Information

Below is the link to the electronic supplementary material.

Supplementary material 1 (PDF 600.9 kb)

Supplementary material 2 (PDF 432.5 kb)

Supplementary material 3 (PDF 286.4 kb)

## Data Availability

The datasets used and/or analysed during the current study are available from the corresponding author on reasonable request.
