# Correction to: Implementation of Guidelines on Family Involvement for Persons with Psychotic Disorders (IFIP): A Cluster Randomised Controlled Trial

**DOI:** 10.1007/s10488-023-01291-w

**Published:** 2023-08-23

**Authors:** Lars Hestmark, Maria Romøren, Kristin Sverdvik Heiervang, Kristiane Myckland Hansson, Torleif Ruud, Jūratė Šaltytė Benth, Irene Norheim, Bente Weimand, Reidar Pedersen

**Affiliations:** 1 Centre for Medical Ethics, University of Oslo, Postbox, Blindern, Oslo, 1130, 0318 Norway; 2 Division of Mental Health Services, Akershus University Hospital, Lørenskog, Norway; 3 Center for Mental Health and Substance Abuse, Faculty of Health and Social Sciences, University of South-Eastern Norway, Drammen, Norway; 4 Institute of Clinical Medicine, University of Oslo, Oslo, Norway; 5 Institute of Clinical Medicine, University of Oslo, Campus Ahus, Oslo, Norway; 6 Health Services Research Unit, Akershus University Hospital, Lørenskog, Norway; 7 Division of Mental Health and Addiction, Vestre Viken Hospital Trust, Lier, Norway; 8 Faculty of Health Sciences, OsloMet Oslo Metropolitan University, Oslo, Norway

**Correction to: Administration and Policy in Mental Health and Mental Health Services Research (2023) 50:520–533**

10.1007/s10488-023-01255-0

The original version of the article unfortunately contained a mistake in Table 3, which was introduced during formatting. The correct version of Table 3 is given below.

**Table 3:** Mean changes and between-group differences in changes with 95% CIs. Results of post hoc analysis from linear mixed models and tobit regression model

Interval	Experimental arm		Control arm		Experimental vs. Control arm		
	Mean change (95% CI)	p-value	Mean change (95% CI)	p-value	Mean change (95% CI)	p-value	Cohen’s d (95% CI)
**BFIS mean**							
0–12	1.01 (0.74; 1.28)	< 0.001					
0–18	1.30 (1.03; 1.57)	< 0.001					
0–24	1.52 (1.25; 1.79)	< 0.001	0.11 (-0.16; 0.38)	0.407	1.41 (1.03; 1.79)	< 0.001	3.41 (1.69; 5.13)
12–18	0.29 (0.02; 0.56)	0.033					
12–24	0.52 (0.25; 0.79)	< 0.001					
18–24	0.22 (-0.05; 0.49)	0.106					
**BFIS-S mean**							
0–12	1.71 (1.44; 1.98)	< 0.001					
0–18	1.93 (1.66; 2.20)	< 0.001					
0–24	2.26 (1.99; 2.53)	< 0.001	-0.03 (-0.30; 0.24)	0.836	2.29 (1.90; 2.67)	< 0.001	5.40 (3.00; 7.80)
12–18	0.21 (-0.06; 0.48)	0.120					
12–24	0.54 (0.27; 0.81)	< 0.001					
18–24	0.33 (0.06; 0.60)	0.017					
**BFIS-P mean**							
0–12	0.61 (0.29; 0.93)	< 0.001					
0–18	0.95 (0.63; 1.27)	< 0.001					
0–24	1.12 (0.80; 1.44)	< 0.001	0.19 (-0.13; 0.51)	0.245	0.93 (0.48; 1.38)	< 0.001	2.03 (0.70; 3.35)
12–18	0.34 (0.02; 0.66)	0.036					
12–24	0.51 (0.19; 0.83)	0.002					
18–24	0.17 (-0.15; 0.49)	0.310					
**GOI mean**							
0–12	2.12 (1.65; 2.60)	< 0.001					
0–18	2.22 (1.75; 2.69)	< 0.001					
0–24	2.19 (1.72; 2.66)	< 0.001	-0.37 (-0.84; 0.10)	0.125	2.56 (1.89; 3.23)	< 0.001	4.60 (2.49; 6.72)
12–18	0.10 (-0.38; 0.57)	0.687					
12–24	0.07 (-0.41; 0.54)	0.781					
18–24	-0.03 (-0.50; 0.44)	0.901					
**FPE scale mean**							
0–12	1.18 (0.19; 2.17)	0.020					
0–18	1.71 (0.72; 2.70)	0.001					
0–24	1.75 (0.76; 2.74)	0.001	-0.94 (-1.94; 0.05)	0.062	2.69 (1.29; 4.09)	< 0.001	2.16 (0.80; 3.52)
12–18	0.53 (-0.46; 1.52)	0.293					
12–24	0.57 (-0.42; 1.56)	0.260					
18–24	0.04 (-0.95; 1.03)	0.939					
**FPE % mean**							
0–12	10.6 (5.4; 15.8)	< 0.001					
0–18	10.0 (4.8; 15.3)	< 0.001					
0–24	8.7 (3.5; 14.0)	0.001	-1.3 (-6.8; 4.3)	0.653	10.0 (2.4; 17.6)	0.010	1.00 (-0.12; 2.12)
12–18	-0.6 (-5.3; 4.1)	0.812					
12–24	-1.9 (-6.6; 2.8)	0.436					
18–24	-1.3 (-6.0; 3.4)	0.588					

