# Supplementary material for: Implementation of Guidelines on Family Involvement for Persons with Psychotic Disorders (IFIP): A Cluster Randomised Controlled Trial
Source: Adm Policy Ment Health. 2023 Feb 16;50(3):520–33. doi: 10.1007/s10488-023-01255-0 (PMC9934504; doi:10.1007/s10488-023-01255-0)
Supplement: Supplementary file 2 — Supplementary material 2 (PDF 432.5 kb) [file 10488_2023_1255_MOESM2_ESM.pdf]

Supplementary file 2. Timeline for the experimental clusters (n =7) of the IFIP trial.

**Activity | Month**	0[1]	1	2	3	4	5	6	7	8	9	10	11	12	13	14	15	16	17	18	19	20	21	22	23	24	25
Fidelity measurements	X	X											X	X					X	X					X	X
Randomisation			X																							
Kick-off sessions					X	X																				
Supervision and training days							X	X							X	X							X	X		
FPE training					X	X	X	X																		
FPE supervision every 6th week									X	X	X	X	X	X	X	X	X	X	X	X	X	X	X	X	X	
Network conferences											X						X					X				
FPE refresher training																X	X	X								
**External factors**																										
Sars-COV-2 Pandemic																	X	X	X	X	X	X	X	X	X	X

[1] November 2018.
