# Supplementary material for: Implementation of Guidelines on Family Involvement for Persons with Psychotic Disorders (IFIP): A Cluster Randomised Controlled Trial
Source: Adm Policy Ment Health. 2023 Feb 16;50(3):520–33. doi: 10.1007/s10488-023-01255-0 (PMC9934504; doi:10.1007/s10488-023-01255-0)
Supplement: Supplementary file 3 — Supplementary material 3 (PDF 286.4 kb) [file 10488_2023_1255_MOESM3_ESM.pdf]

Supplementary file 3. Results of linear mixed models and tobit regression model for difference in change between the experimental and control arm, adjusted for stratification variable.

	**BFIS mean**[b]		**BFIS-S mean**[b]		**BFIS-P mean**[b]		**GOI mean**[b]		**FPE scale mean**[b]		**FPE % mean**[c]	
	RC (SE)	p-value	RC (SE)	p-value	RC (SE)	p-value	RC (SE)	p-value	RC (SE)	p-value	RC (SE)	p-value
Intercept	2.39 (0.18)	<0.001	1.79 (0.19)	<0.001	2.72 (0.20)	<0.001	1.90 (0.21)	<0.001	3.29 (0.45)	<0.001	3.20 (3.74)	0.393
Group[a]	0.22 (0.21)	0.300	0.09 (0.22)	0.694	2.29 (0.24)	0.217	0.04 (0.27)	0.876	-0.14 (0.55)	0.805	3.06 (3.84)	0.424
T12 x Group	1.01 (0.14)	**<0.001**	1.71 (0.14)	**<0.001**	0.61 (0.16)	**<0.001**	2.12 (0.24)	**<0.001**	1.18 (0.52)	**0.022**	10.62 (3.29)	**0.001**
T18 x Group	1.30 (0.14)	**<0.001**	1.93 (0.14)	**<0.001**	0.95 (0.16)	**<0.001**	2.22 (0.24)	**<0.001**	1.71 (0.52)	**0.001**	10.05 (2.43)	**<0.001**
T24	0.11 (0.14)	0.406	-0.03 (0.15)	0.835	0.19 (0.16)	0.244	-0.37 (0.24)	0.128	-0.94 (0.52)	0.067	-1.06 (2.86)	0.711
T24 x Group	1.41 (0.19)	**<0.001**	2.29 (0.19)	**<0.001**	0.93 (0.23)	**<0.001**	2.56 (0.34)	**<0.001**	2.69 (0.73)	**<0.001**	9.81 (4.36)	**0.024**
Stratum												
1	-0.19 (0.22)	0.384	-0.20 (0.23)	0.369	-0.18 (0.24)	0.450	0.06 (0.22)	0.780	-0.01 (0.44)	0.978	2.61 (6.16)	0.672
2 – ref.	0		0		0		0		0		0	
3	-0.27 (0.222)	0.215	-0.27 (0.23)	0.240	-0.28 (0.24)	0.247	-0.50 (0.22)	0.019	-1.45 (0.44)	0.001	-10.20 (3.29)	0.002

[a] Control group – reference; [b] Linear mixed model; [c] Tobit regression model for longitudinal outcome
